# Survival of *Staphylococcus aureus* in Pasteurized White Cheese Preserved Under Different Storage Conditions and Brine Concentrations

**DOI:** 10.1155/ijfo/8481773

**Published:** 2025-12-12

**Authors:** Rnad Haddad, Murad Al-Holy, Amin N. Olaimat, Hamzah Al-Qadiri, Fajer Al-aittan, Narmeen Al-Awwad, Ala Qatatsheh, Mahmoud Abughoush, Anas Al-Nabulsi

**Affiliations:** ^1^ Department of Clinical Nutrition and Dietetics, Faculty of Applied Medical Sciences, The Hashemite University, Zarqa, Jordan, hu.edu.jo; ^2^ Department of Nutrition and Food Technology, School of Agriculture, The University of Jordan, Amman, Jordan, ju.edu.jo; ^3^ Science of Nutrition and Dietetics Program, College of Pharmacy, Al Ain University, Abu Dhabi, UAE, aau.ac.ae; ^4^ Department of Nutrition and Food Technology, Faculty of Agriculture, Jordan University of Science and Technology, Irbid, Jordan, just.edu.jo

**Keywords:** growth, *pathogen*, salt, *shelf-life preservation*, storage temperature

## Abstract

Contamination of brined white cheese (BWC) with pathogenic bacteria is a major concern in the Mediterranean region. This study aimed to investigate the survival capability of *Staphylococcus aureus* in white cheese immersed into different brine solutions (0.0%, 2.5%, 5.0%, 7.5%, and 10.0%) under different temperatures. A cocktail of three strains of *S. aureus,* were inoculated at *ca*. 5 log_10_ CFU/g in BWC. The cheese was stored for 30 days at 4°C, 10°C, or 24°C. The water activity and salt content in cheese ranged between 0.99% and 0.0% in un‐brined cheese to 0.96% and 5.2% in the BWC immersed in 10.0% NaCl. The initial pH of cheese was *ca*. 6.6 and it dropped significantly (*p* < 0.05) after 30 days, especially under high storage temperature. *S. aureus* was able to survive in BWC at 4°C throughout the storage period at counts higher than 3.8 log CFU/g. While at 10°C, the counts remained higher than 6.0 log CFU/g. In comparison, at 24°C, *S. aureus* reached to *ca.* 9.5 log CFU/g after 15 days of storage. TMC (total mesophilic bacterial count) and yeast and mold counts were significantly (*p* < 0.05) lower at higher brine concentrations (7.5% and 10.0%) and lower temperature (4°C). However, the TMC and yeast and mold counts reached their maximum levels after 10 and 5 days when BWC was stored at 10°C and 24°C, respectively. *S. aureus* can survive well in BWC preserved in various brines and different storage temperatures. However, the counts were particularly lower when BWC was kept refrigerated and immersed into 10% brine, emphasizing the necessity of keeping BWC refrigerated and necessitating exploring appropriate preventive measures to reduce the potential risk associated with this pathogen.

## 1. Introduction

Cheese, and in particular, brined white cheese (BWC), is a very popular food product in the Middle East and the Mediterranean region [1, 2]. It is a rennet‐coagulated cheese that can be consumed either fresh or after brining in a brine solution (5%–20%) and storing at room temperature [3]. White cheese can easily become contaminated, especially if stored at abusive temperatures or prepared with poor hygienic conditions [4]. White cheese falls under high water activity (a_w_) foods; containing considerable amounts of fat and protein, and has no competing starter culture; which plays an important role for the growth of pathogenic bacteria, such as *Listeria monocytogenes*, *Escherichia coli* O157:H7, and *Staphylococcus aureus* [2, 5].

*S. aureus* is considered an opportunistic and commensal bacterium, and one of the most common foodborne pathogens [4, 6]. *S. aureus* can cause simple to severe skin infections and sometimes severe cases such as sepsis and bacteremia [4]. *S. aureus* growth and enterotoxin production are affected by several factors, including food storage environment, temperature, pH, salt, and a_w_ [7]. *S. aureus* can survive in a wide range of environments, including low pH (4.2), wide storage temperature (7°C–48°C), and able to tolerate high salt concentrations up to 15%. It is also well known for its capability to produce heat‐resistant toxins that can tolerate heating, freezing, and drying conditions [8]. Staphylococcal food poisoning (SFP) is characterized by nausea, vomiting, abdominal cramps, and diarrhea. The ability of *S. aureus* to produce toxins is triggered by food being stored at 10°C–45°C in the presence of oxygen, pH in the range of 5.3–7.0, a_w_ > 0.86, and in the presence of oxygen [9].

Raw milk and cheese are considered major sources of *S. aureus* strains that may carry genes encoding a variety of enterotoxins that are responsible for SFP [10]. Silva et al. [11] reported that Minas Frescal cheese, a Brazilian type of cheese (pH = 5.8–6.6, salt—1.7%), supports the growth of *S. aureus* and allows the production of enterotoxins at storage temperatures of > 5°C, resulting in a potential cause of food poisoning outbreaks.

Several factors could contribute to cheese contamination with *S. aureus* before, during, or after cheese processing [12]. Milk can be contaminated with *S. aureus* at any step of the food chain from containers and food handlers. Subsequently, when milk is handled without pasteurization, improper processing, and storage conditions, insufficient starter culture activity, and pre‐post–pasteurization contamination, may lead to *S. aureus* growth and toxin production in cheese [13]. The contamination of cheese with *S. aureus* could also be attributed to post‐processing contamination, from using contaminated tools and equipment such as cutting knives, cutting boards, cheesecloth, or preparation of cheese on dirty contact surfaces, and storing the cheese under improper storage conditions [14]. Furthermore, processing factories may be contaminated with *S. aureus* from the floor, packing materials, cheese vats, curd cutters, production room air, cold storage rooms, and brine solutions when making BWC, or through food handlers, especially when food products are frequently touched by hands, this particularly because *S. aureus* is part of normal human microbiota [8, 15, 16].

Many factors that affect the survival of *S. aureus* when present in cheese, for instance, cheese pH, salt concentrations, temperature–time combination during storage period, and a_w_ [5]. Recently, *S. aureus* has been detected in different imported cheese varieties in the United States [17]. Oliveira et al. [14] indicated that *S. aureus* was present in 61% of Brazilian milk and 100% of cheese samples. In November 1988 through January 1989, in England, a SFP outbreak occurred, and it was associated with consuming Stilton cheese made from unpasteurized cow’s milk [18]. Another *S. aureus* outbreak was reported on October 1, 2014 in Switzerland, which was related to soft cheese made from raw milk [19]. *S. aureus* was detected in 142 out of 350 dairy products in Egypt, with positive rates of up to 41% [20].

Because of the outstanding capability of *S. aureus* to live and proliferate in salty foods, it is crucial to explore the growth dynamics of the organism in white cheese preserved in brine. Therefore, the objective of the present study was to investigate the growth behavior of *S. aureus* in pasteurized BWC under different storage temperatures (4°C, 10°C, and 24°C). Additionally, the effect of brine concentration (0.0%, 2.5%, 5.0%, 7.5%, and 10.0%) on the growth pattern of *S. aureus*, total mesophilic bacterial count (TMC), and yeast and mold counts for a 30‐day storage period was also investigated.

## 2. Materials and Methods

### 2.1. Preparation of Pasteurized White Cheese

The BWC was prepared according to the process described by Al‐Nabulsi et al. [12]. Fresh whole fat cow’s milk was used to make white cheese. The full‐fat cow milk was obtained from dairy farms in the Northern part of Jordan and cheese was prepared at the Jordan University of Science and Technology cheese factory. Firstly, the milk was pasteurized by holding at 72°C for 15 s and then cooling the milk to 36°C–37°C. Thereafter, rennet enzyme was added, thus rendering the milk protein casein to coagulate into a curd for 40 min. Thereafter, the whey was drained for 20 min, followed by pressing the resulting curd for 30–60 min at room temperature. Afterward, the pressed curd was cut into cubes (10–15 g), then transfer the cheese into brine solution and then storing at 4°C incubators. A screening test was done to test for the presence of *S. aureus.*

### 2.2. Growth of Microorganisms in BWC

#### 2.2.1. Bacterial Strains and Culture Preparation

The bacterial strains used in the current study were obtained from the Food Microbiology Laboratory, the Hashemite University (Zarqa‐Jordan). Three different strains of *S. aureus* (ATCC 3300, ATCC 13565, and ATCC 33591) were used. *S. aureus* cultures were maintained as frozen stocks at −20°C in 15% glycerol. Three strains were used to have a more representative growth behavior under the different brine concentrations and the different storage conditions.

A stationary‐phase culture of a cocktail of *S. aureus* was used to inoculate the pasteurized white cheese. Bacterial cells in the stationary phase of growth were used because they exhibit the highest resistance to preservation stresses compared with other stages [21]. After thawing, each culture was revived by streaking a loopful on Mannitol Salt agar (MSA) (Oxoid, Basingstoke, Hampshire). The plates were incubated aerobically for 24–48 h at 37°C. The following step involves growing one colony from each isolate separately in brain‐heart infusion (BHI) broth (Oxoid, Basingstoke, Hampshire) and incubating at 37°C for 18–24 h. Then, the fresh cultures were subject to centrifugation for 20 min at 4000 g and washed three times with 10 mL of peptone water solution to prepare for inoculation [21].

#### 2.2.2. Bacterial Inoculation and Submersion of Cheese Into Brine Solution

The manufactured cheese was immersed into sterile brine solutions that were prepared by dissolving appropriate amounts of NaCl in distilled water. Brine solutions at different concentrations were prepared (0.0%, 2.5%, 5.0%, 7.5%, and 10.0%) and sterilized at 121°C for 15 min. Approximately, 150 g portions of the white cheese were kept in a sterile stomacher bag containing 200 mL of sterile brine solution. Thereafter, a cocktail mixture of *S. aureus* was diluted in 0.1% buffered peptone water and then inoculated into the brine solution to make up an approximate initial number of 1.0 × 10^5–6^ CFU/g of cheese. The inoculated white cheese samples were separated and incubated at 4°C, 10°C, and 24°C incubators. The growth behavior of *S. aureus* was monitored for 30 days at several time intervals (0, 1, 5, 10, 15, 20, 25 and 30 days).

#### 2.2.3. Microbiological Analysis

The growth patterns of *S. aureus*, TMC, and yeast and mold were determined in BWC during the 30‐day storage period at the different storage temperatures. Serial dilutions were prepared by aseptically homogenizing a cross‐sectioned 5 g piece of pasteurized white cheese sample with 45 mL of peptone water and shaking for 1 min in a sterile stomacher bag using a stomacher (Model 400.Seward Ltd., London, United Kingdom). *S. aureus* was enumerated by surface plating of 100 *μ*L of a proper dilution of the cheese sample on MSA and incubating aerobically for 48 h at 37°C. In the cases, when *S. aureus* cells were not detectable by direct plating, an enrichment step in a double strength BHI was performed, followed by direct planting onto MSA. For the determination of TMC, proper dilutions of cheese samples were spread‐plated onto Tryptone Soy Agar (TSA, Oxoid, Basingstoke, Hampshire) plates, followed by incubating aerobically at 37°C for 48 h. The yeast and mold numbers were determined by plating 100 *μ*L of properly diluted samples onto Sabouraud Dextrose Agar (SDA) and incubating under aerobic conditions for 3 days at 25°C.

### 2.3. Measurement of Physiochemical Properties of BWC

#### 2.3.1. Water Activity Determination

The a_w_ of cheese samples for all brined cheese treatments containing different salt concentrations at all temperatures were assessed using a water activity meter (Novasina AG, Labmasters a_w_, Lachen, Switzerland) by analyzing approximately 2 g from a cross‐section of BWC samples at 25°C at the beginning and at the end of the storage time. Samples were analyzed in triplicate at room temperature.

#### 2.3.2. pH Determination of Cheese

The pH of cheese samples for all brined cheese treatments containing different salt concentrations at all storage temperatures were assessed using the pH meter (Adwa pH meter, 1000 CE, Adwa, Romania) initially (Day 0) and finally (after 30 days) of storage. The pH electrode was immersed in the BWC until the reading became stable upon which pH values were taken. Samples were analyzed in triplicate at room temperature.

#### 2.3.3. Salt Determination

For the determination of NaCl in cheese, the AOAC method [22] was used, which consists of the determination of chlorides by potentiometric titration with 0.05 N silver nitrate and adding drops of 0.5 N potassium chromate as an indicator. Three grams of pummelled cheese sample was weighed and mixed with 30 mL of distilled water at 55°C. Then, 2 mL of HNO_3_ (in 0.5 mL aliquots) was added and titrated with AgNO_3_. Samples were analyzed in triplicate. The salt concentration was calculated by using the following equation:

%NaCl=V×N×5.84Sample weight

### 2.4. Statistical Analysis

A one‐way ANOVA was performed to examine the effects of various treatment parameters. Each reported value is the mean of three independent experiments, and all values are presented as means ± standard deviations. Paired‐samples *t*‐test was used to compare the means of two variables for a single group. The Statistical Package for the Social Sciences (SPSS) Version 25.0, a program for quantitative analysis of data, was used to examine the data results. A *p* value of < 0.05 was considered significant.

## 3. Results and Discussion

### 3.1. Growth Behavior of *S. aureus* Inoculated in Pasteurized White Cheese Immersed Into Different Brine Solutions and Stored for 30 Days at Different Storage Temperatures

Tables 1, 2, and 3 show the growth behavior of *S. aureus* inoculated into BWC immersed into different concentrations of brine and stored at 4°C, 10°C, and 24°C, respectively. The initial counts of *S. aureus* in the control (cheese in 0.0% brine) and the other brine concentrations were ca. 5.0 log CFU/g. At 4°C, the counts increased significantly (*p* < 0.05) in BWC immersed into the 0.0% (control), 2.5%, and 5.0% brine, and remained constant in cheese immersed into 7.5% and 10.0% brine. After that, *S. aureus* numbers gradually decreased in all treatments to reach 3.3 log CFU/g in control and 3.9–4.2 log CFU/g in cheeses immersed in 2.5%–10% brine (Table 1). Moreover, a noticeable reduction in the counts of *S. aureus* after 15 days of storage was observed, especially for the control cheese. However, *S. aureus* persisted more preferably in the cheese immersed into 2.5% and 5.0% brine solutions. Moreover, at 10°C, *S. aureus* counts increased gradually from day 1 to day 10 to reach 7.1 in cheese immersed 10% brine and to 8.1 log CFU/g in control. Nonetheless, the counts of *S. aureus* in cheese immersed in 10% brine after 20 days of storage at 10°C were significantly higher (*p* < 0.05) compared with cheeses immersed in 0% (control), 2.5%, 5.0%, and 7.5% salt concentration (Table 2). The growth of *S. aureus* at 24°C is presented in Table 3. *S. aureus* increased gradually to > 9.0 log CFU/g in BWC immersed in the control and the different brine concentrations after 5–15 days of storage. However, *S. aureus* counts decreased significantly afterwards and reached to undetectable levels in the control by the end of the 30‐day storage period. It was noticed that the bacterial inactivation increased as the salt concentration decreased where the *S. aureus* counts were 2.3, 4.8, 5.4, and 4.6 log CFU/g in cheeses immersed in 2.5%, 5.0%, 7.5%, and 10.0% brine, respectively. This reduction could be explained by the more acidic environment of the cheese that evolved toward the end of the storage time. Normally, *S. aureus* is a poor competitor microorganism [23] and in the absence of NaCl or in the presence of low brine concentration (2.5%), non‐*S. aureus* bacteria would have resulted in reducing the pH and inhibiting *S. aureus* growth. It was reported that a pH of 5.1 does not support the growth of *S. aureus*, especially when the food a_w_ is less than 0.96 [24].

**Table 1:** Growth of *S. aureus* (log CFU/g) in brined white cheese immersed in different brine solutions and stored for 30 days at 4°C.

**Time (days)**	**Brine concentrations (%)**				
	**0**	**2.5**	**5**	**7.5**	**10**
0	5.09 ± 0.35^bA^	5.16 ± 0.4^bcA^	5.07 ± 0.93^bcA^	5.08 ± 0.32^abA^	4.86 ± 0.22^abA^
1	5.87 ± 0.28^aA^	6.00 ± 0.12^aA^	5.73 ± 0.37^aA^	5.34 ± 0.49^aA^	4.87 ± 0.52^abB^
5	4.65 ± 0.60^bcA^	5.00 ± 0.13^cA^	5.05 ± 0.69^bcA^	4.59 ± 0.26^bA^	4.68 ± 0.17^bA^
10	4.79 ± 0.18^bcAB^	5.00 ± 0.20^cA^	4.70 ± 0.10^bcAB^	4.76 ± 0.15^bAB^	4.49 ± 0.15^bB^
15	4.57 ± 0.23^bcB^	5.46 ± 0.29^bA^	5.63 ± 0.64^abA^	5.34 ± 0.35^aA^	5.41 ± 0.42^aA^
20	4.37 ± 0.45^cAB^	4.83 ± 0.17^cdA^	4.82 ± 0.35^bcA^	4.88 ± 0.08^abA^	3.89 ± 0.29^cB^
25	3.75 ± 0.05^dB^	4.49 ± 0.23^deA^	4.53 ± 0.06^cA^	3.98 ± 0.27^cB^	3.74 ± 0.36^cB^
30	3.34 ± 0.10^dB^	4.15 ± 0.20^eA^	4.21 ± 0.11^cA^	3.87 ± 0.08^cB^	3.85 ± 0.02^cB^

*Note:* Means in the same column with different lowercase letters are significantly different (*p* < 0.05). Means in the same row with different uppercase letters are significantly different (*p* < 0.05). Data represent means ± standard deviations of three measurements.

**Table 2:** Growth of *S. aureus* (log CFU/g) in brined white cheese immersed in different brine solutions and stored for 30 days at 10°C.

**Time (days)**	**Brine concentrations (%)**				
	**0**	**2.5**	**5**	**7.5**	**10**
0	5.09 ± 0.35^dA^	5.16 ± 0.41^dA^	5.07 ± 0.93^dA^	5.08 ± 0.32^eA^	4.86 ± 0.22^dA^
1	5.62 ± 0.35^cAB^	5.80 ± 0.47^bcdA^	4.97 ± 0.34^dBC^	4.93 ± 0.54^dBC^	4.46 ± 0.41^dC^
5	5.84 ± 0.23^cA^	6.21 ± 0.05^bA^	6.03 ± 0.46^cA^	6.02 ± 0.07^cA^	5.91 ± 0.09^cA^
10	8.08 ± 0.13^aA^	7.89 ± 0.03^aAB^	7.70 ± 0.20^aB^	7.65 ± 0.18^aB^	7.10 ± 0.24^abC^
15	7.12 ± 0.62^bA^	7.44 ± 0.85^aA^	6.94 ± 0.30^bA^	6.75 ± 0.63^bA^	7.43 ± 0.68^aA^
20	6.14 ± 0.12^cB^	6.22 ± 0.46^bB^	6.33 ± 0.40^bcB^	6.63 ± 0.15^bcAB^	7.09 ± 0.17^abA^
25	5.91 ± 0.17^cB^	5.42 ± 0.19^cdC^	5.93 ± 0.15^cB^	6.51 ± 0.12^bcA^	6.78 ± 0.10^Ba^
30	6.00 ± 0.14^cC^	6.02 ± 0.05^bcC^	6.31 ± 0.06^bcB^	6.34 ± 0.04^bcB^	6.76 ± 0.12^bA^

*Note:* Means in the same column with different lowercase letters are significantly different (*p* < 0.05). Means in the same row with different uppercase letters are significantly different (*p* < 0.05). Data represent means ± standard deviations of three measurements.

**Table 3:** Growth of *S. aureus* (log CFU/g) in brined white cheese immersed in different brine solutions and stored for 30 days at 24°C.

**Time (days)**	**Brine concentrations (%)**				
	**0**	**2.5**	**5**	**7.5**	**10**
0	5.09 ± 0.35^dA^	5.16 ± 0.41^cA^	5.07 ± 0.93^dA^	5.08 ± 0.32^cA^	4.86 ± 0.22^cA^
1	6.20 ± 0.10^cA^	6.53 ± 0.23^bA^	6.67 ± 0.22^cA^	6.60 ± 0.20^bA^	6.55 ± 0.08^bA^
5	9.57 ± 0.98^bA^	10.08 ± 0.61^aA^	9.71 ± 0.46^aA^	9.77 ± 0.11^aA^	9.58 ± 0.25^aA^
10	10.53 ± 0.07^aA^	9.80 ± 0.46^aB^	8.62 ± 0.28^bC^	9.36 ± 0.28^aB^	9.46 ± 0.10^aB^
15	9.79 ± 0.59^abA^	9.57 ± 0.28^aA^	9.47 ± 0.31^aA^	9.81 ± 0.55^aA^	9.37 ± 0.41^aA^
20	4.00 ± 0.01^eD^	5.11 ± 0.22^cC^	6.84 ± 0.21^cA^	6.39 ± 0.36^bB^	6.78 ± 0.15^bAB^
25	ND 0/3	3.73 ± 0.37^dC^	4.74 ± 0.03^dA^	4.83 ± 0.02^cA^	4.32 ± 0.10^cB^
30	ND 0/3	2.30 ± 0.43^eC^	4.80 ± 0.30^dB^	5.37 ± 0.27^cA^	4.55 ± 0.17^cA^

*Note:* Means in the same column with different lowercase letters are significantly different (*p* < 0.05). Means in the same row with different uppercase letters are significantly different (*p* < 0.05). Data represent means ± standard deviations of three measurements. ND: not detected (after nonselective enrichment followed by direct plating).

*S. aureus* can tolerate high salt content up to 15% and temperatures between 7°C and 48.5°C [4, 8]. These characteristics help the bacteria to remain viable after 30 days of storage at different temperatures. Pasteurized BWC are widely consumed in the Mediterranean area including Jordan. Therefore, keeping the product refrigerated throughout the entire dairy production chain is crucial to preclude the risk of SFP. Most *S. aureus* strains are able to grow over a wide range of a_w_ (from 0.83 to > 0.99) [25]. Elahi and Fujikawa [7] also reported that increasing NaCl in food to 5% NaCl had little effect on the growth of *S. aureus*, whereas concentrations of 10% to 20% resulted in a noticeable inhibitory effect against the *S. aureus*. Osmoregulators are normally produced in osmotolerant microorganisms such as *S. aureus* to combat osmotic stress [26]. Osmoregulators such as L‐proline are upregulated in *S. aureus* when present in foods with high salinity. Such substances are overproduced to maintain osmotic pressure and protect bacterial intracellular constituents [27]. Nonetheless, it was reported that the viability of *S. aureus* was diminished when the organism is present in a high salinity environment (20% NaCl), which results in damaging cell structure and therefore releasing proline and other constituents outside bacterial cell [27].

### 3.2. Growth Behavior of Total Mesophilic Bacteria in Pasteurized White Cheese Inoculated With *S. aureus* Immersed Into Different Brine Solutions and Stored for 30 Days at Different Storage Temperatures

The growth pattern of TMC in BWC immersed into different brine concentrations and stored at 4°C for 30 days is presented in Table 4. A gradual increase in TMC was noticed in white cheese immersed into different concentration of brine and the magnitude of growth was inversely related to salt concentration. In the control (BWC immersed in sterile distilled water), the TMC increased from an initial count of *ca*. 6.0 logs at Day 0 and reached to *ca.* 8.0 logs after 30 days of storage. However, when immersing BWC into 7.5% and 10.0% brine solution, TMC significantly decreased (*p* < 0.05) by the end of the 30‐day storage time, where the counts reached to ca. 6.1 and 5.3 logs, respectively. In comparison, the counts reached to 7.6 and 7.5, when the cheese was immersed into 2.5% and 5.0% brine solutions. On the other hand, when the cheese was stored at 10°C (Table 5), the brine concentration affected the growth of TMC, especially during the first 5 days of storage and the counts were significantly lower (*p* < 0.05) at higher brine concentrations (7.5% and 10% brine). However, the effect of brine concentration was less pronounced and the TMC increased steadily and reached to counts that exceeded 9.3 logs at all brine concentrations after 10–20 days of storage. After that, the TMC counts decreased significantly (*p* < 0.05) by the end of the 30‐day storage time and reached to ca. 8.5 logs.

**Table 4:** Growth of total mesophilic bacteria (log CFU/g) in brined white cheese inoculated with *S. aureus* immersed into different brine concentrations and stored for 30 days at 4°C.

**Time (days)**	**Brine concentrations (%)**				
	**0**	**2.5**	**5**	**7.5**	**10**
0	5.90 ± 0.08^gB^	5.86 ± 0.08^eB^	6.16 ± 0.17^dA^	6.42 ± 0.10^cA^	6.14 ± 0.05^cA^
1	7.09 ± 0.22^deA^	6.62 ± 0.21^dA^	7.11 ± 0.21^bcA^	7.12 ± 0.39^bA^	6.66 ± 0.35^bcA^
5	6.43 ± 0.20^fgA^	6.44 ± 0.28^dA^	6.77 ± 0.39^cA^	6.26 ± 0.26^cA^	6.05 ± 0.63^dA^
10	8.74 ± 0.35^aA^	8.66 ± 0.34^aA^	8.64 ± 0.66^aA^	8.70 ± 0.30^aA^	7.67 ± 0.35^aB^
15	8.19 ± 0.77^abA^	8.10 ± 0.04^bAB^	7.36 ± 0.39^bcAB^	7.15 ± 0.76^bBC^	7.05 ± 0.15^abC^
20	7.58 ± 0.09^cdA^	7.57 ± 0.13^cA^	7.25 ± 0.06^bcAB^	7.25 ± 0.07^bAB^	7.19 ± 0.36^abB^
25	6.96 ± 0.11^efA^	6.74 ± 0.07^dA^	6.75 ± 0.13^cA^	6.22 ± 0.11^cB^	5.50 ± 0.42^deC^
30	8.12 ± 0.08^bcA^	7.63 ± 0.17^cB^	7.47 ± 0.23^bB^	6.05 ± 0.21^cC^	5.25 ± 0.15^eD^

*Note:* Means in the same column with different lowercase letters are significantly different (*p* < 0.05). Means in the same row with different uppercase letters are significantly different (*p* < 0.05). Data represent means ± standard deviations of three measurements.

**Table 5:** Growth of total mesophilic bacteria (log CFU/g) in brined white cheese inoculated with *S. aureus* immersed into different brine concentrations and stored for 30 days at 10°C.

**Time (days)**	**Brine concentrations (%)**				
	**0**	**2.5**	**5**	**7.5**	**10**
0	5.90 ± 0.08^dC^	5.86 ± 0.08^dC^	6.16 ± 0.17^dB^	6.42 ± 0.10^eA^	6.14 ± 0.05^dB^
1	7.33 ± 0.15^cA^	7.26 ± 0.09^cA^	7.09 ± 0.09^cA^	6.10 ± 0.09^eB^	6.08 ± 0.31^dB^
5	8.56 ± 0.04^bA^	8.60 ± 0.11^bA^	8.39 ± 0.14^bA^	7.17 ± 0.28^dB^	6.93 ± 0.03^dB^
10	9.88 ± 0.23^aA^	9.64 ± 0.18^aA^	9.85 ± 0.22^aA^	10.02 ± 0.2^aA^	9.67 ± 0.41^abA^
15	10.14 ± 0.18^aA^	9.79 ± 0.27^aA^	10.01 ± 0.13^aA^	9.82 ± 0.17^abA^	9.33 ± 1.15^abA^
20	9.95 ± 0.19^aA^	10.12 ± 0.53^aA^	10.03 ± 0.60^aA^	9.42 ± 0.17^bA^	10.10 ± 0.15^aA^
25	9.72 ± 0.66^aA^	10.08 ± 0.51^aA^	9.71 ± 0.59^aA^	9.60 ± 0.58^abA^	8.85 ± 0.20^bcA^
30	8.86 ± 0.23^bA^	8.63 ± 0.23^bA^	8.66 ± 0.21^bA^	8.38 ± 0.42^cA^	8.42 ± 0.46^cA^

*Note:* Means in the same column with different lowercase letters are significantly different (*p* < 0.05). Means in the same row with different uppercase letters are significantly different (*p* < 0.05). Data represent means ± standard deviations of three measurements.

The growth pattern of TMC in BWC stored at 24°C is presented in Table 6. Regardless of brine concentration, the TMC increased significantly (*p* < 0.05) from an initial level of *ca.* 6.0 log CFU/g to more than 10.0 log CFU/g after 5 days of storage and the counts persisted at these high levels till 20 days of storage. However, the counts started to decline afterwards and reached to counts between 8.3 and 9.7 log CFU/g by the end of the storage period.

**Table 6:** Growth of total mesophilic bacteria (log CFU/g) in brined white cheese inoculated with *S. aureus* immersed into different brine concentrations and stored for 30 days at 24°C.

**Time (days)**	**Brine concentrations (%)**				
	**0**	**2.5**	**5**	**7.5**	**10**
0	5.90 ± 0.08^eC^	5.86 ± 0.08^eC^	6.16 ± 0.17^eB^	6.42 ± 0.10^eA^	6.14 ± 0.05^cB^
1	7.56 ± 0.16^dA^	7.77 ± 0.11^dA^	7.58 ± 0.08^dA^	7.26 ± 0.22^dB^	7.25 ± 0.03^b^
5	10.18 ± 0.40^aA^	10.30 ± 0.40^aA^	10.20 ± 0.35^abA^	10.18 ± 0.12^abA^	10.25 ± 0.19^aB^
10	10.10 ± 0.18^aA^	10.33 ± 0.35^aA^	10.32 ± 0.47^abA^	10.08 ± 0.40^abcA^	10.13 ± 0.21^aA^
15	10.10 ± 0.41^aA^	10.20 ± 0.42^aA^	9.78 ± 0.24^cdA^	10.45 ± 0.30^aA^	10.15 ± 0.25^aA^
20	10.17 ± 0.24^aA^	10.27 ± 0.23^aA^	10.47 ± 0.22^aA^	10.15 ± 0.08^abA^	10.19 ± 0.23^aA^
25	9.08 ± 0.11^bD^	9.37 ± 0.17^bC^	9.89 ± 0.19^bcB^	9.86 ± 0.14^bcB^	10.27 ± 0.16^aA^
30	8.29 ± 0.17^cB^	8.64 ± 0.03^cB^	9.73 ± 0.04^cA^	9.63 ± 0.46^cA^	9.74 ± 0.79^aA^

*Note:* Means in the same column with different lowercase letters are significantly different (*p* < 0.05). Means in the same row with different uppercase letters are significantly different (*p* < 0.05). Data represent means ± standard deviations of three measurements.

Generally, the safety and microbial quality of brined cheeses are based on the quality of raw milk, the type of heat treatment of cheese milk, the extent of microbial contamination during processing (especially during salting), the rate of salt absorption, cheese moisture, and pH development during ripening [28]. The ability of bacteria and fungi to survive and thrive in salted cheese habitat depends on many factors including their capability to utilize available energy sources and other nutrients, tolerating low pH conditions, high salt concentrations, and low a_w_ [29]. It is likely that salt‐resistant non‐starter lactic acid bacteria (LAB), mainly *Lactobacilli* and *Enterococci* predominate during storage time [30]. The most common LAB in BWC are mesophilic *Pediococcus* spp.*, Lactobacilli* spp.*, Enterococcus* spp., and *Leuconostoc* spp. [31]. Additionally, LAB may be present in cheese as contaminants from personnel and processing equipment environment [32]. Similar results were obtained by Eljagmani and Altuner [33] who assessed the microbiological quality of the Turkish BWC at 5°C, 15°C, and 25°C and found the TMC ranged from 3.2 × 10^6^ log CFU/g to 1.76 × 10^8^ log CFU/g, and the growth was higher in cheese stored at 25°C compared with lower temperatures. On the other hand, the TMC in BWC kept in 15% brine solution and stored at 12°C for 30 days declined from 9.4 log CFU/g at Day 1 to 4.6 log CFU/g by 30 days of ripening [34].

### 3.3. Growth Behavior of Yeast and Mold in Pasteurized White Cheese Inoculated With *S. aureus* Immersed Into Different Brine Solutions and Stored for 30 Days at Different Storage Temperatures

The growth behavior of yeast and mold in BWC immersed into different concentrations of brine and stored for 30 days at 4°C is shown in Table 7. The yeast and mold increased to high levels at low brine concentrations (≤ 5%) compared with higher concentrations (7.5 and 10.0). A gradual increase in the yeast and mold count was noticed, especially in the control and at a brine concentration of 2.5%. The counts in the control increased from an initial level of ca. 4.0 logs to ca. 8.3 logs by the end of the storage period. Comparable counts were also obtained for the yeast and mold in the BWC immersed into 2.5% brine. After 20 days of storage, the counts for yeast and mold in the cheese stored at brine concentrations < 5%, were significantly higher compared with the counts in cheese at higher brine concentrations (7.5 and 10.0). Nonetheless, when the BWC was stored at 10°C (Table 8), maximum yeast and mold counts in all brine solutions were attained after 10 days of storage, where the numbers reached > 8.0 logs. However, the numbers started to decline gradually thereafter. Yet, the counts remained higher than the initial counts after 30 days of storage. Also, it is noteworthy that the yeast and mold counts in the cheese immersed into 10.0% brine solution were significantly lower (*p* < 0.05) compared with all other brine concentrations after 25 and 30 days of storage at 10°C (Table 8). On the other hand, the growth pattern of yeast and mold in BWC at 24°C is shown in Table 9. The highest counts were obtained after 5 days of storage. Yet, the counts remained high (ca. 8.5 logs) till the end of the storage time in the un‐brined (control) and the cheese brine in the 2.5% solution. However, the counts of yeast and mold declined significantly (*p* < 0.05) in the cheese stored in brine at 5.0%–10.0% concentrations, especially at 10.0% brine concentration, where the count (5.6 log CFU/g) was significantly (*p* < 0.05) lower compared with 5.0%–7.5% brine (6.0–6.4 log CFU/g) by the end of the 30‐day storage time. Nonetheless, it is well reported that *Debaryomyces hansenii* is one of the most salt‐tolerant yeast species that can grow in foods with 16% salt concentrations. This species is repeatedly detected in high salt environments such as brined cheeses [28]. In the current study, yeast and mold counts were particularly lower at higher brine concentration (10%) and lower storage temperatures (4°C and 10°C). This agrees with El Owni et al. [35], who indicated that yeast and mold counts decreased when Domiati cheese was stored under refrigeration and the counts decreased gradually compared with the initial counts and reached to undetectable levels after 120 days of storage under refrigeration.

**Table 7:** Growth of yeast and mold (log CFU/g) in pasteurized white cheese inoculated with *S. aureus* immersed into different brine concentrations and stored for 30 days at 4°C.

**Time (days)**	**Brine concentrations (%)**				
	**0**	**2.5**	**5**	**7.5**	**10**
0	3.85 ± 0.26^dBC^	3.81 ± 0.30^eC^	4.10 ± 0.22^fABC^	4.42 ± 0.03^deAB^	4.22 ± 0.09^bcA^
1	5.32 ± 0.32^cBC^	5.81 ± 0.22^bcAB^	6.05 ± 0.09^bcA^	5.77 ± 0.17^aAB^	4.97 ± 0.48^abC^
5	5.50 ± 0.11^cA^	5.55 ± 0.14^bcA^	5.87 ± 0.40^cA^	5.31 ± 0.40^abA^	4.62 ± 0.54^abB^
10	5.37 ± 0.51^cA^	4.96 ± 0.84^dA^	5.51 ± 0.19^dA^	5.09 ± 0.17^bcA^	4.73 ± 0.34^abA^
15	5.06 ± 0.62^cA^	5.34 ± 0.65^cdA^	5.08 ± 0.11^eA^	4.89 ± 0.19^bcA^	4.69 ± 0.39^abA^
20	5.44 ± 0.20^cA^	5.39 ± 0.49^bcA^	5.20 ± 0.03^deA^	3.91 ± 0.31^eB^	4.05 ± 0.38^cB^
25	6.28 ± 0.09^bA^	6.20 ± 0.11^bA^	6.31 ± 0.19^abA^	4.67 ± 0.70^cdB^	5.08 ± 0.25^aB^
30	8.31 ± 0.49^aA^	8.22 ± 0.02^aB^	6.51 ± 0.09^agC^	5.06 ± 0.09^bcD^	4.79 ± 0.65^abD^

*Note:* Means in the same column with different lowercase letters are significantly different (*p* < 0.05). Means in the same row with different uppercase letters are significantly different (*p* < 0.05). Data represent means ± standard deviations of three measurements.

**Table 8:** Growth of yeast and mold (log CFU/g) in pasteurized white cheese inoculated with *S. aureus* immersed into different brine concentrations and stored for 30 days at 10°C.

**Time (days)**	**Brine concentrations (%)**				
	**0**	**2.5**	**5**	**7.5**	**10**
0	3.85 ± 0.26^eBC^	3.81 ± 0.30^eC^	4.10 ± 0.22^fAB^	4.42 ± 0.03^gA^	4.22 ± 0.09^dAB^
1	6.05 ± 0.19^dA^	5.98 ± 0.03^dA^	5.88 ± 0.06^dA^	5.28 ± 0.12^fB^	5.13 ± 0.24^cB^
5	6.42 ± 0.21^cA^	5.95 ± 0.36^dA^	6.34 ± 0.32^cA^	6.26 ± 0.18^cA^	5.29 ± 0.28^cB^
10	8.04 ± 0.17^aAB^	8.34 ± 0.15^aAB^	8.77 ± 0.24^aA^	8.18 ± 0.21^aB^	8.31 ± 0.39^aAB^
15	7.68 ± 0.06^bA^	7.66 ± 0.07^bA^	7.88 ± 0.25^bA^	6.65 ± 0.33^bB^	6.40 ± 0.17^bB^
20	7.59 ± 0.30^bA^	7.47 ± 0.15^bA^	7.65 ± 0.15^bA^	5.68 ± 0.11^dB^	5.17 ± 0.96^cB^
25	5.86 ± 0.17^dA^	5.95 ± 0.04^dA^	5.46 ± 0.07^eB^	5.73 ± 0.19^dA^	4.33 ± 0.35^dC^
30	6.58 ± 0.15^cA^	6.65 ± 0.16^cA^	6.62 ± 0.11^cA^	5.39 ± 0.14^eB^	4.27 ± 0.20^dC^

*Note:* Means in the same column with different lowercase letters are significantly different (*p* < 0.05). Means in the same row with different uppercase letters are significantly different (*p* < 0.05). Data represent means ± standard deviations of three measurements.

**Table 9:** Growth of yeast and mold (log CFU/g) in pasteurized white cheese inoculated with *S. aureus* immersed into different brine concentrations and stored for 30 days at 24°C.

**Time (days)**	**Brine concentrations (%)**				
	**0**	**2.5**	**5**	**7.5**	**10**
0	3.85 ± 0.26^eB^	3.81 ± 0.30^fB^	4.10 ± 0.22^fAB^	4.42 ± 0.03^fA^	4.22 ± 0.09^gA^
1	6.66 ± 0.08^dAB^	6.72 ± 0.06^eA^	6.56 ± 0.20^deAB^	6.37 ± 0.28^eBC^	6.15 ± 0.05^eC^
5	10.13 ± 0.69^aA^	10.03 ± 0.27^aA^	10.03 ± 0.45^aA^	10.13 ± 0.13^aA^	10.18 ± 0.19^aA^
10	9.22 ± 0.49^bA^	9.63 ± 0.25^abA^	9.45 ± 0.21^bA^	9.27 ± 0.19^bA^	9.35 ± 0.46^bA^
15	9.47 ± 0.73^abA^	9.04 ± 0.96^bcA^	9.16 ± 0.39^bA^	8.81 ± 0.53^bA^	9.00 ± 0.26^bA^
20	8.18 ± 0.35^cA^	7.80 ± 0.73^dA^	8.32 ± 0.18^cA^	7.56 ± 0.24^cA^	7.71 ± 0.24^cA^
25	6.37 ± 0.64^dA^	6.32 ± 0.28^eA^	7.03 ± 0.28^dA^	6.98 ± 0.26^dA^	6.64 ± 0.45^dA^
30	8.32 ± 0.05^cA^	8.50 ± 0.07^cdA^	6.38 ± 0.10^eB^	5.98 ± 0.25^eC^	5.61 ± 0.19^fD^

*Note:* Means in the same column with different lowercase letters are significantly different (*p* < 0.05). Means in the same row with different uppercase letters are significantly different (*p* < 0.05). Data represent means ± standard deviations of three measurements.

### 3.4. Salt Content, Water Activity, and Changes in pH of BWC Inoculated With *S. aureus*

The current study revealed that salt (NaCl) content of BWC ranged between 0.0 in the un‐brined chees to *ca.* 5.2% in the cheese immersed in 10.0% brine concentration and the a_w_ values ranged between 0.99 and 0.96 in the BWC immersed in 0.0% and the 10.0% brine solutions, respectively. As it is evident in Table 10 that the pH of BWC was storage temperature‐dependent and the pH of the cheese decreased as the storage temperature increased from 4°C to 24°C. The initial pH values of the BWC ranged from 6.3 to around 7.0. A significant reduction in the pH of cheese immersed in distilled water from 7.08 to 6.08, 6.99 to 5.30, and 6.68 to 5.15 after 30 days of storage were noticed at 4°C, 10°C, and 24°C, respectively. It was evident that as the storage temperature increases, the magnitude of reduction in pH concomitantly increases. Likewise, BWC immersed into 2.5%, 5.0%, 7.5%, and 10.0% brine solutions exhibited a slight reduction in pH values when stored at 4°C compared with BWC stored at 10°C and 24°C. In general, the greatest reduction of pH was mostly observed at 24°C. This result agrees with the results obtained by Al‐Nabulsi et al. [5] who reported that the pH values of white cheese immersed into 10% and 15% brine concentrations and stored for 28 days at 10°C and 25°C exhibited the maximum diminution in pH values compared with the cheese stored at 4°C. The same study indicated that the pH declined from and initial pH of 6.72 to 6.30 and 5.91 at 10°C and 24°C, respectively, in cheese kept in 10% brine solution; while it dropped from 6.72 to 5.94 and 5.32 at 24°C when immersed in 15% brine solution.

**Table 10:** Changes in pH of brined white cheese inoculated with *Staphylococcus aureus* and immersed in different brine solutions and stored for 30 days at different storage temperatures.

**Temperature (°C)**	**Brine concentration%**	**Initial**	**Final**
4	0.0	7.08 ± 0.02^A^	6.08 ± 0.12^A^
	2.5	6.64 ± 0.02^A^	6.47 ± 0.02^B^
	5.0	6.57 ± 0.00^A^	6.37 ± 0.01^B^
	7.5	6.56 ± 0.06^A^	6.36 ± 0.01^B^
	10.0	6.51 ± 0.04^A^	6.33 ± 0.02^B^

10	0.0	6.99 ± 0.04^A^	5.30 ± 0.01^A^
	2.5	6.64 ± 0.03^A^	4.95 ± 0.06^A^
	5.0	6.64 ± 0.06^A^	4.86 ± 0.06^A^
	7.5	6.45 ± 0.01^A^	5.15 ± 0.04^A^
	10.0	6.35 ± 0.03^A^	5.71 ± 0.06^A^

24	0.0	6.68 ± 0.14^A^	5.15 ± 0.07^A^
	2.5	6.37 ± 0.04^A^	5.49 ± 0.02^A^
	5.0	6.39 ± 0.01^A^	5.78 ± 0.01^A^
	7.5	6.38 ± 0.04^A^	5.12 ± 0.00^A^
	10.0	6.32 ± 0.01^A^	4.92 ± 0.05^A^

*Note:* Means with the same uppercase capital letter are not significantly different (*p* > 0.05). Data represent means ± standard deviations of three measurements.

## 4. Conclusions

BWC is a popular food product consumed on a large scale in the Mediterranean region. It is a high a_w_ food with relatively neutral pH and bountiful amounts of nutrients that could serve as an important medium for the colonization of bacteria, such as *S. aureus*. The current study demonstrated that *S. aureus* was capable of surviving in different brine concentrations for the 30‐day storage period under different storage temperatures. In contrast to lower storage temperatures, *S. aureus* growth was noticeably greater at 24°C. The bacterium survived well into BWC stored at 4°C, but it grew more preferably at abusive refrigeration (10°C) and room temperatures (24°C). This indicates distributing or storing BWC under abusive storage temperature could underpin the growth of *S. aureus* and compromise the safety of the product. Additionally, the microbial quality of the BWC deteriorated more rapidly at abusive (10°C) and room temperature (24°C) conditions, especially, when the cheese was immersed in low brine concentrations. Also, *S. aureus* grew more favorably when lower brine concentrations were used (≤ 5.0%). The microbial quality of the product deteriorated more rapidly at higher storage temperatures (10°C and 24°C) and at lower brine concentrations as revealed by the high TMC and yeast and mold counts. The results obtained in the current study guarantee using higher brine concentrations (10% brine) and keeping the food refrigerated to restrict the growth of *S. aureus.* As the pathogen was also capable of surviving in 10% brine and at 4°C, it is prudent to include appropriate intervention approaches to further restrain the growth of *S. aureus*.

## Disclosure

All authors read and approved the final manuscript.

## Conflicts of Interest

The authors declare no conflicts of interest.

## Author Contributions

The study conception and design, material preparation, data collection and analysis were performed by Rnad Haddad (R.H.), Murad Al‐Holy (M.A‐H.), and Amin N. Olaimat (A.N.O.). Data analysis and revising initial draft were contributed by Murad Al‐Holy, Rnad Haddad, and Amin N. Olaimat. Hamzah Al‐Qadiri, Fajer Al‐aittan, Narmeen Al‐Awwad, Ala Qatatsheh, Mahmoud Abughoush, and Anas Al‐Nabulsi contributed to conceptualization, writing and final revisions of the manuscript.

## Funding

This project was financially supported by the Deanship of Scientific Research and Faculty of Graduate Studies at The Hashemite University, Zarqa, Jordan.

## Data Availability

Data available on request from the authors.
